# Correction: Toxicity of tributyltin to the European flat oyster *Ostrea edulis*: Metabolomic responses indicate impacts to energy metabolism, biochemical composition and reproductive maturation

**DOI:** 10.1371/journal.pone.0341792

**Published:** 2026-01-28

**Authors:** The PLOS One Staff

## Notice of republication

This article was republished on January 13, 2026, to correct an error in the first author’s name. The correct name is: Lina M. Zapata-Restrepo. The correct citation is: Zapata-Restrepo LM, Hauton C, Hudson MD, Williams ID, Hauton D (2023) Toxicity of tributyltin to the European flat oyster *Ostrea edulis*: Metabolomic responses indicate impacts to energy metabolism, biochemical composition and reproductive maturation. PLoS One 18 (2): e0280777. https://doi.org/10.1371/journal.pone.0280777

## Supporting information

S1 File Originally published, uncorrected article. (PDF)

S2 File Republished, corrected article. (PDF)
