# Supplementary material for: Toxicity of tributyltin to the European flat oyster Ostrea edulis: Metabolomic responses indicate impacts to energy metabolism, biochemical composition and reproductive maturation
Source: PLoS One. 2023 Feb 6;18(2):e0280777. doi: 10.1371/journal.pone.0280777 (PMC9901812; doi:10.1371/journal.pone.0280777)
Supplement: S1 Fig — IC-MS/MS was performed using a ICS-5000+ HPLC system, C18 reversed-phase analysis of underivatised samples was performed using a Thermo Utimate 3000 UHPLC system, and C18 reversed-phase analysis of derivatised samples was also performed using the Thermo Utimate 3000 UHPLC system coupled directly to a Q-Exactive HF Hybrid Quadrupole-Orbitrap mass spectrometer. Models are assessed by cross validation (using R2, Q2 and Accuracy; R2 should be higher than Q2 which should be >0.4 and within 0.3 if the R2 values, the closer to 1 the R2 and Q2 the strong the model). Partial Least Squares-Discrimination Analysis (PLSDA) indicated a high degree of correlation between the modelled data and the predicted analysis for TBTCl exposure. Furthermore, permutation test analysis indicated that the results were significantly different (p<0.01 for all) implying the data were not overfitted. (DOCX) [file pone.0280777.s001.docx]

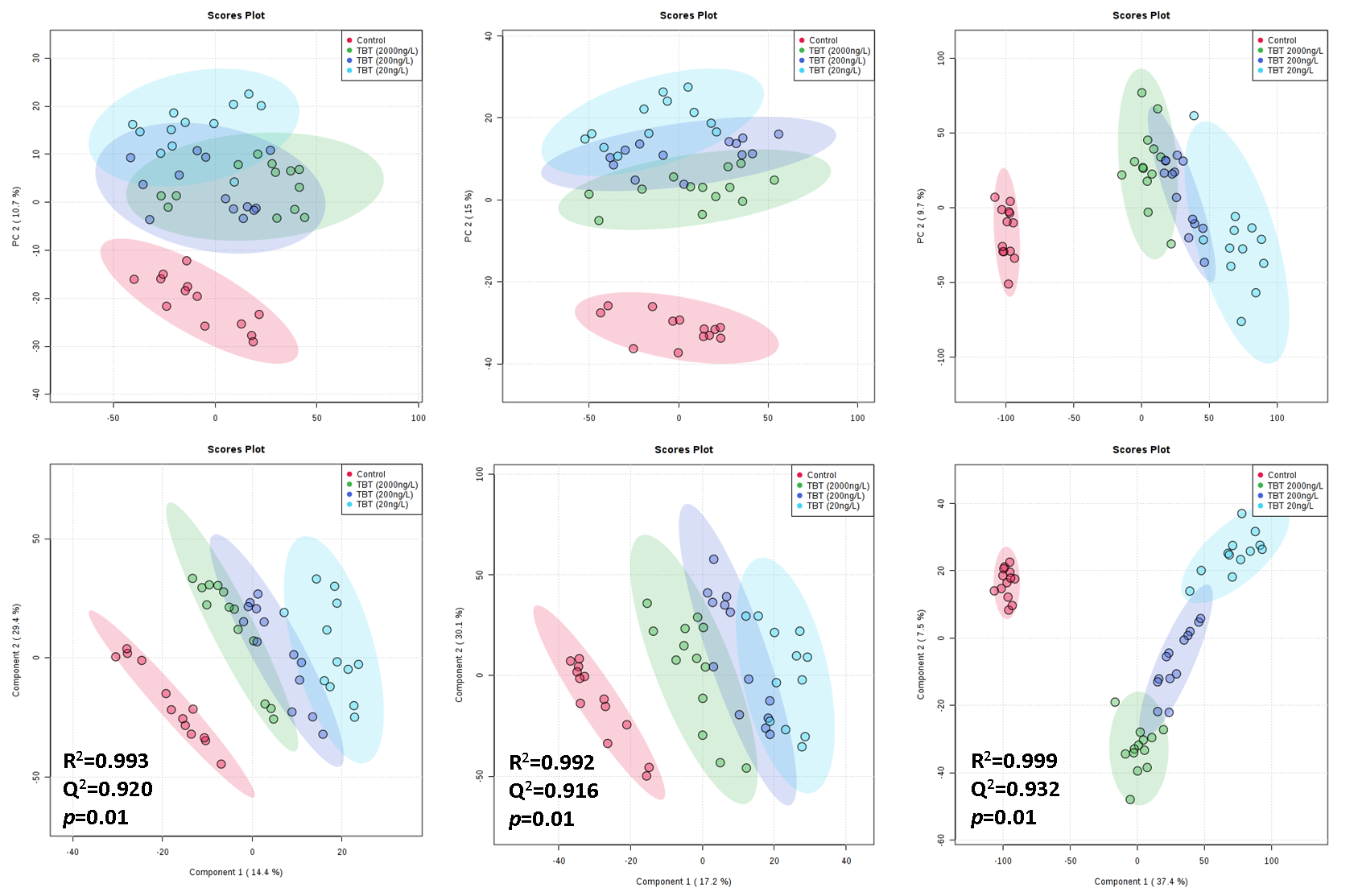


**S1 Figure**. Principal component analysis and partial least square discriminant analysis model comparing *Ostrea edulis* metabolomic profiles among TBTCl treatments: 20 ng/L (n=13), 200 ng/L (n=13) and 2000 ng/L (n=13), and a negative control (n=13) after 9 weeks of exposure. IC-MS/MS was performed using a ICS-5000+ HPLC system, C18 reversed-phase analysis of underivatised samples was performed using a Thermo Utimate 3000 UHPLC system, and C18 reversed-phase analysis of derivatised samples was also performed using the Thermo Utimate 3000 UHPLC system coupled directly to a Q-Exactive HF Hybrid Quadrupole-Orbitrap mass spectrometer. Models are assessed by cross validation (using R2, Q2 and Accuracy; R2 should be higher than Q2 which should be >0.4 and within 0.3 if the R2 values, the closer to 1 the R2 and Q2 the strong the model). Partial Least Squares-Discrimination Analysis (PLSDA) indicated a high degree of correlation between the modelled data and the predicted analysis for TBTCl exposure. Furthermore, permutation test analysis indicated that the results were significantly different (p<0.01 for all) implying the data were not overfitted.
