# Supplementary material for: Toxicity of tributyltin to the European flat oyster Ostrea edulis: Metabolomic responses indicate impacts to energy metabolism, biochemical composition and reproductive maturation
Source: PLoS One. 2023 Feb 6;18(2):e0280777. doi: 10.1371/journal.pone.0280777 (PMC9901812; doi:10.1371/journal.pone.0280777)
Supplement: S1 Table — (DOCX) [file pone.0280777.s002.docx]

**S1 Table.** *Ostrea edulis* biometric parameters (mean±SD) for total Weight (W), Height (H), length (L), width (Wi), Shell volume (SVol), flesh weight (FW) and Condition Index (CI) at the beginning of the experiment (t0), under different treatments (20 ng/L, 200 ng/L and 2000 ng/L of TBTCl), and a negative control after 9 weeks

| **Treatment** | **n** | **W (g)** | **H (mm)** | **L (mm)** | **Wi (mm)** | **SVol (ml)** | **FW (g)** | **CI** |
| --- | --- | --- | --- | --- | --- | --- | --- | --- |
| **t0** | 6 | 71.66±10.99 | 77.20±3.00 | 72.02±7.05 | 19.33±4.22 | 48.37±9.71 | 8.29±2.03 | 11.49±1.83 |
| **20 ng/L** | 13 | 63.30±7.05 | 70.56±10.43 | 71.22±4.92 | 20.96±3.04 | 45.46±6.12 | 6.83±0.80 | 10.86±1.27 |
| **200 ng/L** | 13 | 63.26±11.23 | 75.01±3.32 | 70.66±4.49 | 21.94±2.43 | 38.51±6.03 | 6.64±1.11 | 10.81±2.76 |
| **2000 ng/L** | 13 | 68.44±9.75 | 71.35±4.17 | 71.87±3.89 | 19.42±2.45 | 38.97±8.07 | 6.93±1.72 | 10.05±1.44 |
| **Control** | 13 | 65.51±10.09 | 73.51±5.46 | 71.44±3.93 | 20.00±2.50 | 35.91±6.63 | 7.41±1.18 | 11.40±1.75 |
