# Supplementary material for: Toxicity of tributyltin to the European flat oyster Ostrea edulis: Metabolomic responses indicate impacts to energy metabolism, biochemical composition and reproductive maturation
Source: PLoS One. 2023 Feb 6;18(2):e0280777. doi: 10.1371/journal.pone.0280777 (PMC9901812; doi:10.1371/journal.pone.0280777)
Supplement: S2 Table — (DOCX) [file pone.0280777.s003.docx]

**S2 Table.** Gonad development stage*s* classified as inactive (G0), early gametogenesis (G1), advanced gametogenesis (G2), ripe gonad (G3), partially spawned gonad (G4) and reabsorbing gonad (G5) according to the sex categories (females and males) identified at the end of the exposure to TBTCl

| **Treatment** | **Gonad development stage** | | | | | | | | | |
| --- | --- | --- | --- | --- | --- | --- | --- | --- | --- | --- |
|  | **Females** | | | | | **Males** | | | | |
|  | G0 | G1 | G2 | G3 | G4 | G0 | G1 | G2 | G3 | G4 |
| Control |  | 3 | 4 |  |  |  | 2 |  |  |  |
| 20 ng/L |  | 4 |  |  |  |  | 2 |  |  | 1 |
| 200 ng/L |  | 2 |  | 1 |  |  | 4 |  |  | 1 |
| 2000 ng/L |  |  | 3 |  |  |  |  |  |  |  |
