# Supplementary material for: Correction: Toxicity of tributyltin to the European flat oyster Ostrea edulis: Metabolomic responses indicate impacts to energy metabolism, biochemical composition and reproductive maturation
Source: PLoS One. 2026 Jan 28;21(1):e0341792. doi: 10.1371/journal.pone.0341792 (PMC12851448; doi:10.1371/journal.pone.0341792)
Supplement: S1 File — (PDF) [file pone.0341792.s001.pdf]

PLOS ONE

RESEARCH ARTICLE

Toxicity of tributyltin to the European flat oyster *Ostrea edulis*: Metabolomic responses indicate impacts to energy metabolism, biochemical composition and reproductive maturation

Lina M. Zapata-Restrepio[1☯]*, Chris Hauton[2], Malcolm D. Hudson[1], Ian D. Williams[3], David Hauton[4☯]

1 School of Geography and Environmental Sciences, University of Southampton, Highfield Campus, Southampton, United Kingdom, 2 Ocean and Earth Sciences, National Oceanography Centre, University of Southampton, Southampton, Hampshire, United Kingdom, 3 Faculty of Engineering and Physical Sciences, University of Southampton, Highfield Campus, Southampton, United Kingdom, 4 Metabolomics Research Group, Chemistry Research Laboratory, University of Oxford, Oxford, United Kingdom

☯ These authors contributed equally to this work.
* lmzr1f20@soton.ac.uk

Check for updates

OPEN ACCESS

Citation: Zapata-Restrepio LM, Hauton C, Hudson MD, Williams ID, Hauton D (2023) Toxicity of tributyltin to the European flat oyster *Ostrea edulis*: Metabolomic responses indicate impacts to energy metabolism, biochemical composition and reproductive maturation. PLoS ONE 18(2): e0280777. https://doi.org/10.1371/journal.pone.0280777

Editor: Michael Schubert, Laboratoire de Biologie du Développement de Villefranche-sur-Mer, FRANCE

Received: August 16, 2022

Accepted: January 10, 2023

Published: February 6, 2023

Copyright: © 2023 Zapata-Restrepio et al. This is an open access article distributed under the terms of the Creative Commons Attribution License, which permits unrestricted use, distribution, and reproduction in any medium, provided the original author and source are credited.

Data Availability Statement: All relevant data are within the paper and its Supporting Information files. However, the same data content in the Supporting Information are held in a public repository of the University of Southampton. DOI: https://doi.org/10.5258/SOTON/D2465

Funding: This work and Zapata-Restrepo were supported by The University of Southampton

Abstract

Tri-Butyl Tin (TBT) remains as a legacy pollutant in the benthic environments. Although the toxic impacts and endocrine disruption caused by TBT to gastropod molluscs have been established, the changes in energy reserves allocated to maintenance, growth, reproduction and survival of European oysters *Ostrea edulis*, a target species of concerted benthic habitat restoration projects, have not been explored. This study was designed to evaluate the effect of TBT chloride (TBTCl) on potential ions and relevant metabolomic pathways and its association with changes in physiological, biochemical and reproductive parameters in *O. edulis* exposed to environmental relevant concentrations of TBTCl. Oysters were exposed to TBTCl 20 ng/L (n = 30), 200 ng/L (n = 30) and 2000 ng/L (n = 30) for nine weeks. At the end of the exposure, gametogenic stage, sex, energy reserve content and metabolomic profiling analysis were conducted to elucidate the metabolic alterations that occur in individuals exposed to those compounds. Metabolite analysis showed significant changes in the digestive gland biochemistry in oysters exposed to TBTCl, decreasing tissue ATP concentrations through a combination of the disruption of the TCA cycle and other important molecular pathways involved in homeostasis, mitochondrial metabolism and antioxidant response. TBTCl exposure increased mortality and caused changes in the gametogenesis with cycle arrest in stages G0 and G1. Sex determination was affected by TBTCl exposure, increasing the proportion of oysters identified as males in *O. edulis* treated at 20ng/l TBTCl, and with an increased proportion of inactive stages in oysters treated with 2000 ng/l TBTCl. The presence and persistence of environmental pollutants, such as TBT, could represent an additional threat to the declining *O. edulis* populations and related taxa around the world, by increasing mortality, changing reproductive maturation, and disrupting metabolism. Our

Faculty of Engineering and the Environment Graduate School and the Colombia Government scholarship (Administrative Department of Science, Techonology and Innovation (Departamento Administrativo de Ciencia, Tecnología e Innovación - COLCIENCIAS), PhD scholarship, call 646 (2014). The funders had no role in study design, data collection and analysis, decision to publish, or preparation of the manuscript

Competing interests: The authors have declared that no competing interests exist.

findings identify the need to consider additional factors (e.g. legacy pollution) when identifying coastal locations for shellfish restoration.

Introduction

Organotin compounds were extensively used in a variety of industrial products including antifouling paints for boats [1–3], causing considerable damage to localized coastal areas before being banned by the International Maritime Organisation (IMO) in 2008. The most well-known organotin, tributyltin (TBT), is highly persistent in the environment [4–7] and is the most toxic compound known to aquatic ecosystems [1] mainly due to its high lipid solubility that provides easy cell penetration, facilitating cell absorption [1] with significant bioaccumulation over time. The introduction of legislation to reduce TBT inputs from vessels and the establishment of restrictions for using TBT as an anti-fouling paint occurred in the late 1980s and successive bans were proposed since then [8, 9]. Studies in the decade after the 1987 TBT restrictions showed a reduction in concentrations of this pollutant in water and organisms [10, 11]. However, TBT remains a widespread global contaminants and several years after the IMO legislation organotin compounds such as Monobutyltin trichloride (MBTC), Dibutyltin dichloride (DBTC), tributyltin chloride (TBTCl), diphenyltin dichloride (DphTC), and triphenyltin chloride (TphTC) persists in some estuaries, ports and harbours [12–14]. Recently, water samples from areas with international shipping and maritime recreation activities still showed values of TBT above the threshold levels (e.g. in the Hamble Estuary in Southampton Water, England; [11, 15]). TBT is strongly adsorbed to organic matter and sediments retain concentrations of TBT for long periods and it can be released to the water column when sediment is disturbed, for example by dredging or during storm events [1, 2, 5].

The accumulation of TBT and its metabolites have been observed in mussels and oysters [16–19]. Moreover, changes in growth, biochemical and physiological parameters (e.g. shell thickening) [20–22] and toxicity at low concentrations of TBT for adult marine bivalves [22–24] have been reported. *O. edulis* has been shown to be sensitive to concentrations of TBTCl as low as 10 ng/L in seawater, exhibiting significant digestive cell atrophy correlated with autophagic mechanisms, catabolic metabolism, reduced bioenergetic balances and reduced somatic growth [22]. There have been few reports of the effect of TBTCl on bivalves suggesting decrease in ATP synthesis [25] and TCA cycle disturbance [26] as some of the underlying molecular mechanisms of action of this compound. However, the association between TBTCl, these metabolic changes and effects on reproduction on *O. edulis* still remains unclear.

Ostrea edulis, the largest oyster native to Europe, is found from the low intertidal down to the sublittoral zone throughout the Atlantic and Mediterranean coasts of Europe [27–29]. *O. edulis* populations are ecologically and economically valuable but populations in Europe have suffered several collapses during the last century [30–35]. Additionally, a skewed sex ratio toward male phase oysters in members of the family Ostreidae, including the *O. edulis*, has been reported [36–40]. Some of these studies have reported oyster populations in a good condition in terms of growth, survival, and immune function but with male-biased sex ratio [36, 40]. A cyclically skewed sex ratio, especially a male-biased sex ratio, could decrease the effective breeding population size [41] making the populations susceptible to other external factors that precipitate population declines. Whilst a number of factors have been attributed to this declines [28–29, 34], the presence of chemical pollutants in the aquatic environment potentially adds an additional environmental stress causing the reduction of natural populations

worldwide [42]. It is thus important to understand if pollution by TBT could be one of the factors that may trigger or affect sex changes in this species.

The effect of TBT on molluscs reproduction and, specifically, its masculinization effect have been extensively reported in gastropods, causing imposex, sterility and ultimately affecting its reproductive capability [43–46]. However, due to the complexity and variety of hermaphroditism strategies in bivalve molluscs changes are more nuanced and a reproductive effect caused by organotin compounds is more difficult to understand. Ovarian spermatogenesis, female-dominated populations resulting from the inhibition or delay in the normal switch from male to female, and effects on larvae production have been shown to be affected by organotins in different bivalve species [24, 46–49]. However, the changes in investment of energy resources and metabolic pathways affected as a consequence of TBTCl exposure during gonadal development have not been explored.

Therefore, this study aimed to investigate the changes in metabolite profile and metabolomic pathways and associated changes in physiological, biochemical and reproductive parameters in *O. edulis* exposed to environmentally-relevant concentrations of TBTCl. With reintroduction schemes for *O. edulis* around Europe and beyond [29, 50] and on-going dredging work potentially disturbing the TBT stored in the sediment in ports located close to important restoration areas [13, 51–53], understanding the impacts of TBTCl on this species should be a priority.

Results

Tri-butyl tin chloride (TBTCl) exposure is associated with disruptions in multiple metabolic pathways

Exploiting multiple metabolomic platforms we demonstrate that metabolic disruption in the digestive gland of *O. edulis* is associated with TBTCl exposure. Analyses demonstrated that TBTCl exposure was associated with changes in a broad range of ion features by all three of the liquid chromatography (LC) methods used [54]. The exploitation of three methods of liquid chromatography-mass spectrometry (LC-MS) allowed for the detection of the broadest range of metabolites and is evidenced by the subsequent detection of some 349 identified compounds. Ion Exchange LC-MS detected 5,733 ion features, with 1773 showing coefficient of variation (CV) $<30\%$ (and hence well-characterised); from these 157 compounds were identified with reference to authenticated standards. For C18-reverse phase LC-MS, 10103 ion features were detected, with 2577 features showing CV$<30\%$ and of these 147 compounds were identified. For the derivatised-C18-reverse phase LC-MS, detecting primary and secondary amines, 16027 ion features were detected, with 10940 features with CV$<30\%$ and 58 compounds being identified.

Principal Component Analysis (PCA) of raw data from ion exchange, C18-reverse phase and derivatised-C18-reverse phase chromatography all indicated strong separation between control tissue and TBTCl-exposed tissue. Interestingly, for all methods overlap was noted between all groups exposed to TBTCl irrespective of concentration, suggesting common changes as a consequence of TBTCl exposure (S1 Fig).

Energy metabolism and reserves. Estimates of adenine nucleotide concentrations indicated that at 2000ng/L TBTCl, total adenine nucleotides (TAN) in the digestive tissue declined 50% ($p<0.05$; Fig 1A). Direct analysis of individual adenine nucleotides showed that both 200ng/L and 2000ng/L TBTCl exposure was associated with a significant increase in the percentage of TAN present as ATP ($p<0.05$ for both; Fig 1B) and significantly decreased ADP levels by one-third ($p<0.05$ for both; Fig 1B). In addition, TBTCl exposure increased the proportion of TAN as AMP ($p<0.01$; for both; Fig 1B) at 200ng/L and 2000ng/L TBTCl.

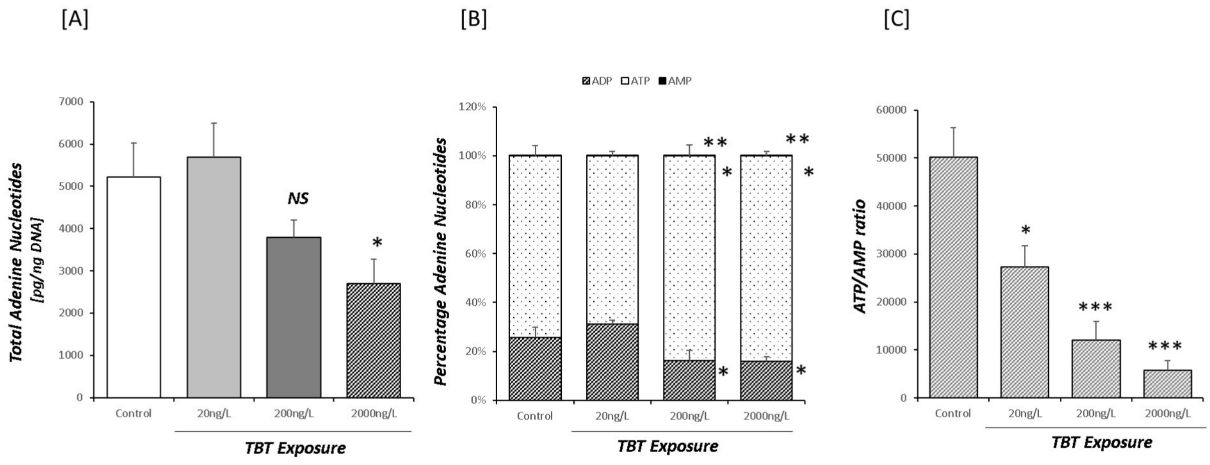

Fig 1. Estimates of (A) total, (B) percentage of individual adenine nucleotide concentrations and (C) ATP/AMP ratio in *Ostrea edulis* exposed to 20 ng/L (n = 13), 200 ng/L (n = 13) and 2000 ng/L (n = 13) of TBTCl for 9 weeks.

https://doi.org/10.1371/journal.pone.0280777.g001

The degree of metabolic stress within a tissue or organism can be estimated by quantifying the ATP/AMP concentration ratio. We observed an dose-dependent inverse relationship between the concentration of TBTCl and the ATP/AMP ratio. This was a function of decreasing ATP concentrations coupled with increased AMP concentrations. Following exposure to 20ng/L TBTCl the ATP/AMP ratio halved ($p<0.05$; Fig 1C), for 200ng/L TBTCl the ATP/AMP ratio decreased by 80% ($p<0.001$; Fig 1C) with 2000ng/L TBTCl decreased the ATP/AMP ratio by 90% ($p<0.001$; Fig 1).

Free fatty acids (FFA) represent stored sources of oxidative energy, derived from nutrients consumed. Tissue levels of certain FFA were significantly decreased with TBTCl exposure; with palmitic (40%), stearic (40%), linoleic (60%) and oleic acids (40%) reduced following TBTCL exposure at all doses ($p<0.001$; Fig 2A–2C and 2F). By contrast, tissue levels of decandoic, phytanic and hydroxyoctanoic acids as well as stearoylcarnitine were unchanged by TBTCL exposure (NS for all; Fig 2D, 2E, 2G and 2H).

Krebs cycle metabolites. Mapping the fold-change for selected metabolites onto specific metabolic pathways indicated that some molecules involved in the TCA cycle may be a primarily impacted by exposure to TBTCl. Decreases in methyl-isocitrate and oxaloacetate ($p<0.001$) (Fig 3) were noted following exposure to 2000ng/L TBTCl. TBTCl exposure was also associated with a depletion of fumarate at all concentrations ($p<0.001$; Fig 3E).

Digestive tissue retinoids. TBTCl exposure was associated with a significant decrease in tissue retinol concentrations in digestive gland, with both 20ng/L and 200ng/L TBTCl decreasing tissue levels 5-fold ($p<0.001$ for both; Fig 4A). However, at the highest TBTCL concentration (2000ng/L) retinol levels were restored. In contrast, 9/13-cis-retinoic acid showed a dose-dependent increase. At 200ng/L TBTCl 9/13-cis-retinoic increased 1.2-fold ($p<0.05$; Fig 4B), with 2000ng/L recording increases of 1.25 ($p<0.05$; Fig 4B). Tissue All-Trans Retinoic Acid (ATRA) was unaffected by TBTCl exposure (NS for all doses; Fig 4C), however retinyl-ester levels in digestive gland were decreased in a dose-dependent manner and halved at the high dose (2000ng/L $p<0.01$; Fig 4D).

Antioxidant defense. Exposure to 200ng/L TBTCl was associated with a 1.2-fold increase in reduced glutathione levels ($p<0.05$; Fig 5A); by contrast both low (20ng/L) and high dose (2000ng/L) TBTCL had no effect on GSH concentration (NS for both; Fig 5A). Oxidised glutathione (GSSG) was significantly decreased by 60% following exposure to TBTCl at all

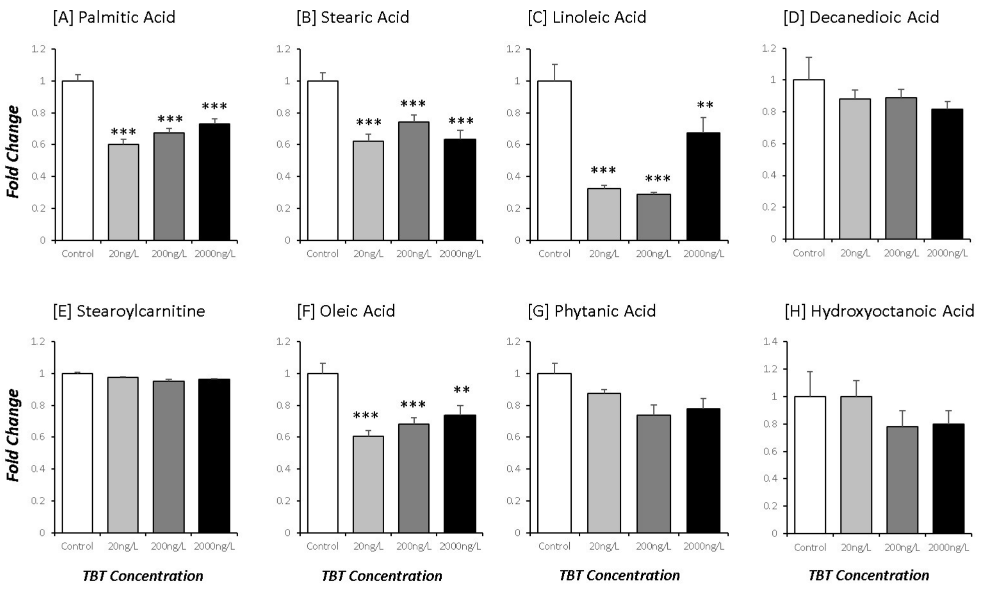

Fig 2. Fold-change of (A) palmitic acid, (B) stearic acid, (C) linoleic acid, (D) decanedioic acid, (E) stearoylcarnitine, (F) oleic acid, (G) phytanic acid, and (H) hydroxyoctanoic acid in *Ostrea edulis* exposed to three different concentrations of TBTCl: 20 ng/L (n = 13), 200 ng/L (n = 13) and 2000 ng/L (n = 13), and a negative control (n = 13) kept under laboratory conditions for 9 weeks.

https://doi.org/10.1371/journal.pone.0280777.g002

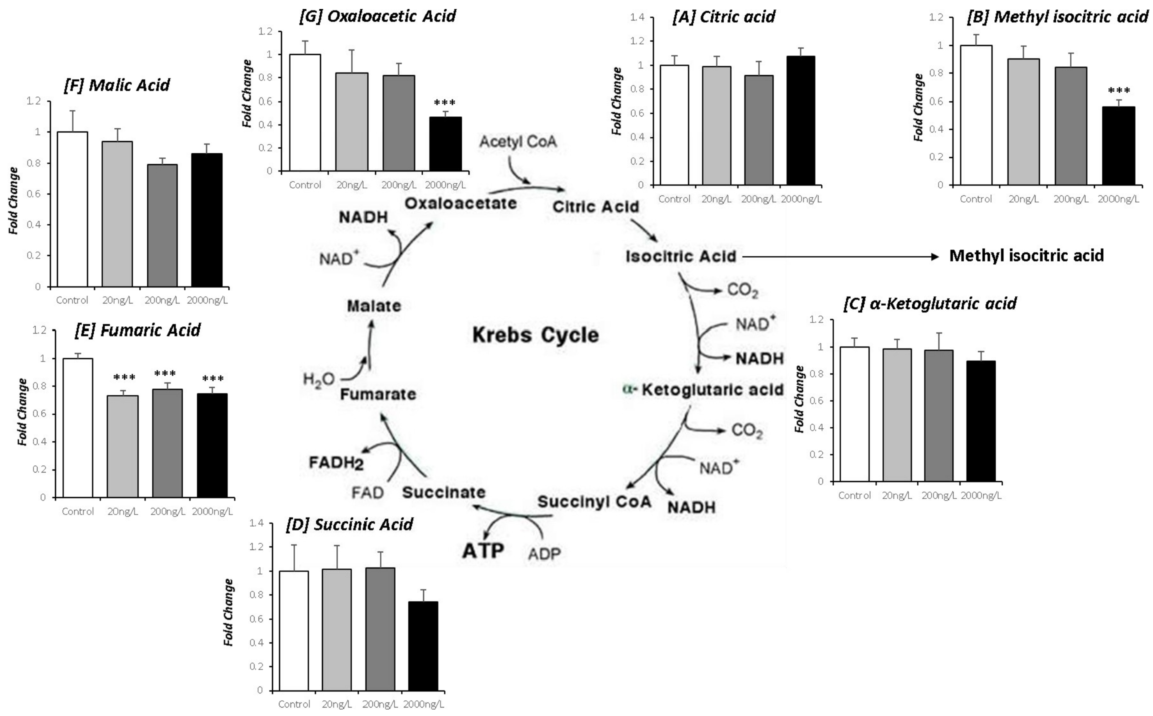

Fig 3. Fold-changes for (A) Citric acid, (B) Methyl isocitric acid, (C) a-ketoglutaric acid, (D) Succinic acid, (E) Fumaric acid, (F) Malic acid, and (G) Oxaloacetic acid from the TCA cycle in *Ostrea edulis* exposed to three different concentrations of TBTCl: 20 ng/L (n = 13), 200 ng/L (n = 13) and 2000 ng/L (n = 13), and a negative control (n = 13) kept under laboratory conditions for 9 weeks.

https://doi.org/10.1371/journal.pone.0280777.g003

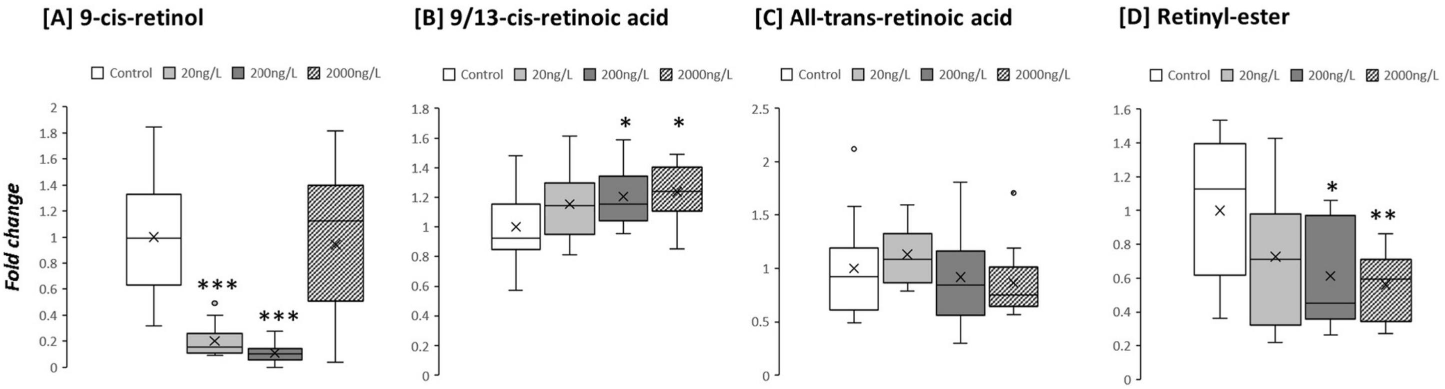

Fig 4. Fold-change of retinoids in *Ostrea edulis* exposed to three different concentrations of TBTCl: 20 ng/L (n = 13), 200 ng/L (n = 13) and 2000 ng/L (n = 13), and a negative control (n = 13) kept under laboratory conditions for 9 weeks.

https://doi.org/10.1371/journal.pone.0280777.g004

concentrations (p<0.01 for all; Fig 5B). To quantify the redox status of digestive gland tissue the ratio of GSH/GSSG was calculated. For untreated tissue the GSH/GSSG ratio was estimated at 2.1. Following exposure to low-dose TBTCl (20ng/L) this ratio doubled (p<0.05; Fig 5C). Further increasing TBTCl concentration (200ng/L) increased the glutathione ratio to 5 (P<0.01; Fig 5C) and TBTCl levels at 2000ng/L increased the glutathione ratio to 9 (p<0.01; Fig 5C).

Digestive gland gross biochemical composition

To understand if changes in metabolite profile were associated with changes in the biochemical composition of digestive gland of *O. edulis*, gross biochemical composition was determined at the end of the experiment. Exposure to TBTCL at 200ng/L and 2000ng/L led to a 2-fold increase in tissue lipid (p<0.001; Fig 6). Tissue lipid concentration was highest (55.60±5.21% DW) in the 200 ng/L TBTCl treatment, followed by treatments with 2000 ng/L (46.73±3.81% DW) and 20 ng/L (30.31±0.89%DW). Tissue protein also increased following exposure to TBTCl, with 2-fold higher protein noted at 200ng/L TBTCl and 2.5-fold higher at 2000ng/L (p<0.001 for both; Fig 6). However, carbohydrate concentration decreased after exposure to the highest concentration of TBTCl (p<0.05; Fig 6) but did not show any effect at the other two treatments.

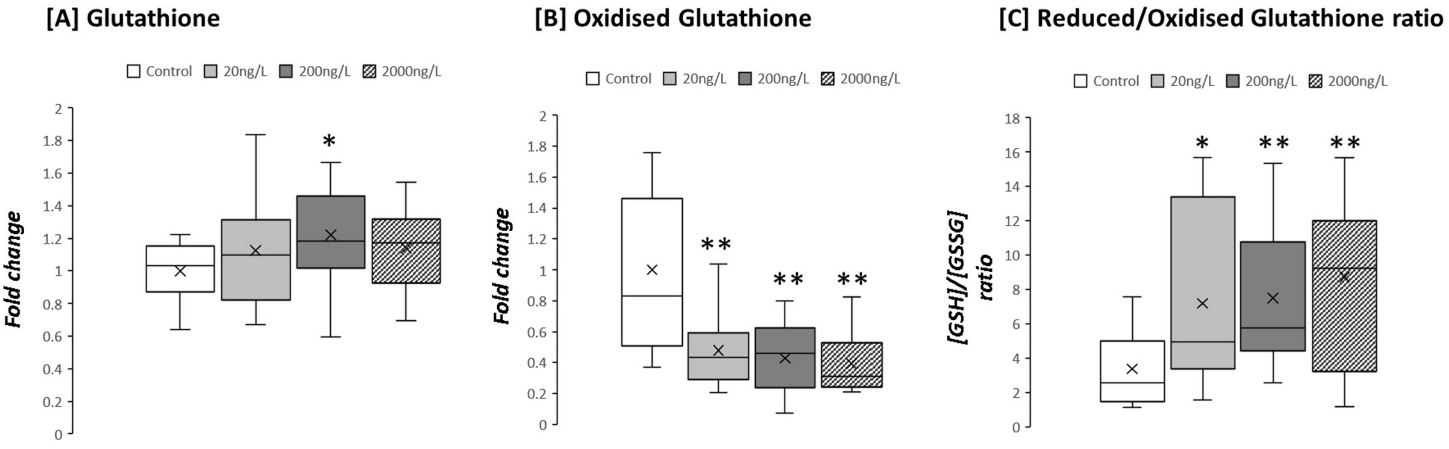

Fig 5. Fold-change of glutathione levels in *Ostrea edulis* exposed to three different concentrations of TBTCl: 20 ng/L (n = 13), 200 ng/L (n = 13) and 2000 ng/L (n = 13), and a negative control (n = 13) kept under laboratory conditions for 9 weeks.

https://doi.org/10.1371/journal.pone.0280777.g005

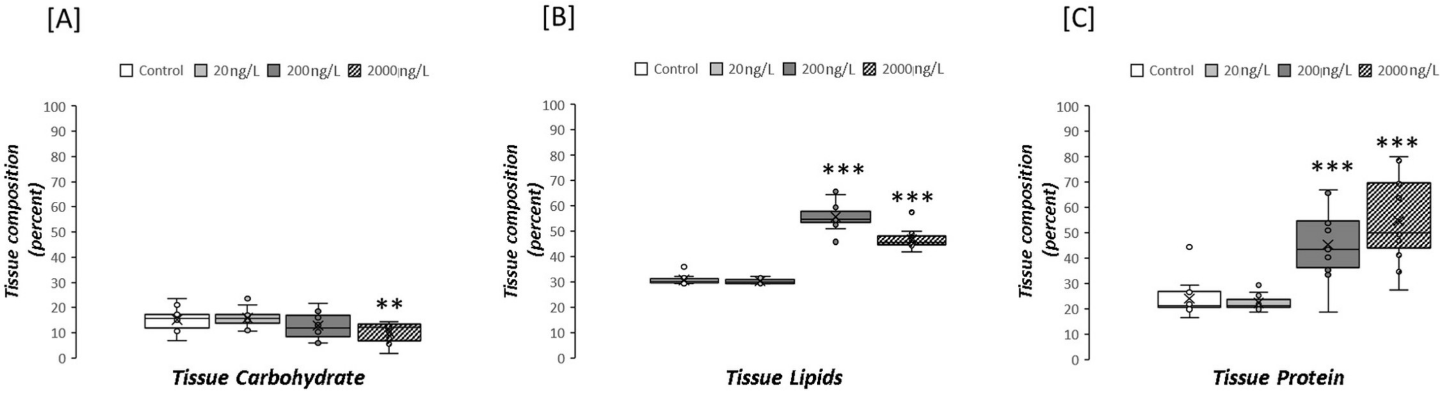

Fig 6. Total lipids (% of dry weight, % DM), total carbohydrates (% of dry weight, % DM) and protein content (% of dry weight, % DM) in gonads of *Ostrea edulis* exposed 20 ng/L (n = 13), 200 ng/L (n = 13) and 2000 ng/L (n = 13) of TBTCl, and a negative control (n = 13) kept under laboratory conditions for 9 weeks. Error bars denote standard deviation.

https://doi.org/10.1371/journal.pone.0280777.g006

TBTCl exposure caused disrupted gametogenesis in oysters

At the beginning of the experiment, two-thirds of the oysters were classified in gonad stage G1 (early gametogenesis), according to Da Silva et al. [39] and one-third in G2 (advanced gametogenesis), confirming that no gonadal maturation (follicles contained mature gametes such as spermatozoa balls and mature ovocytes) had started before the exposure. By the end of the TBTCl exposure 15% of the control group oysters were classified in stage G0 (considered as inactive,), 62% in stage G1, 15% in stage G2 and 8% in stage G3 (ripe gonad) (Fig 7A) showing a progression in the gonad development as expected. No oysters were found in stages G4 (partially spawned gonad) or G5 (reabsorbing gonad) for the control group. An interruption in gonad development was observed at the end of the experiment for all TBTCl exposure groups (Fig 7A). Exposure to TBTCl increased the proportion of animals classified as inactive, with

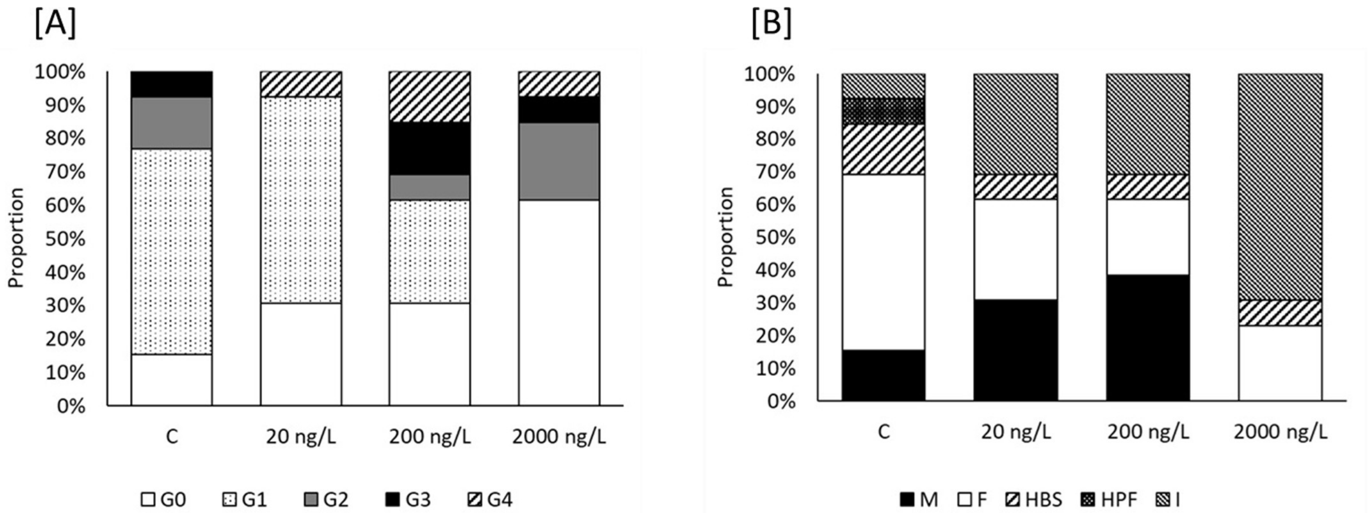

Fig 7. Different stages of gonad development (A) and sex categories (B) of *Ostrea edulis* exposed to three different concentrations of TBTCl: 20 ng/L (n = 13), 200 ng/L (n = 13) and 2000 ng/L (n = 13), and a negative control (n = 13) kept under laboratory conditions for 9 weeks. Developmental stage and sexes were determined according to da Silva et al. [39]. Gametogenic stage of the gonad classified as inactive (G0), early gametogenesis (G1), advanced gametogenesis (G2), ripe gonad (G3), partially spawned gonad (G4) and reabsorbing gonad (G5). Specimens sex was identified by histological examination as male (M), female (F), hermaphrodite with both sexes equally represented (HBS), hermaphrodite predominantly male (HPF), and indeterminate (I).

https://doi.org/10.1371/journal.pone.0280777.g007

33.33%, 30.77% and 69.23% inactive at 20ng/L, 200ng/L and 2000ng/L TBTCl, respectively (Fig 7B). It was not possible to discriminate the effect on gametogenesis respect to the sex categories due to the low number of females and males obtained in some treatments (S2 Table).

To further investigate the effect of TBTCl on sex ratio, we histologically inspected the proportion of males, females and hermaphrodites in *O. edulis* exposed to TBTCl. At the beginning of the experiment 60% of the animals were classified as females, 20% as males, and 20% were determined to have both sexes equally represented (HBS). Exposure to TBTCl caused significant changes ($\chi^2 = 18.27$, df = 1, p = 0.006) in the sex ratio in the experimental groups (Fig 7). For control oysters, 54% were designated as female, 15% males, 8% identified as hermaphrodite-predominantly-male (HPF) and 15% were determined HBS. From the control group, one oyster was designated as inactive. Exposure to TBTCl at 20ng/L and 200ng/L led to a dose-dependent decrease in individuals identified as female, with a concomitant increase in oysters declared male. Following exposure to the highest dose of TBTCl (2000ng/L) 23% presented as female and a further 8% as HBS. Interestingly at 2000 ng/L of TBTCl no males were found.

TBTCl exposure impacts oyster condition, leading to mortality

Oyster biometric parameters of survivors at the end of the experiment were generally unchanged in all groups, irrespective of TBT exposure (S1 Table). However, exposure to 2000ng/L led to a significant decrease in Body Condition Index ($p < 0.05$; Fig 8). Ultimately, a dose-dependent increase in oyster mortality ($p<0.05$) was observed following exposure to TBTCl. At the end of the exposure 7.01% of oysters died in the control group compared to 21.8% at 20ng/L TBTCL, 26.9% at 200ng/L and 34.3% for oysters exposed to 2000ng/L TBTCL ($p < 0.05$, Fisher's exact test) (Fig 8).

Discussion

Tributyltin has been considered to be the most toxic compound to aquatic ecosystems, mainly due to its high lipid solubility that facilitates cell absorption and significant bioaccumulation over time [1]. In this study we sought to establish the potential toxic effect of chronic exposure to TBTCl on the metabolism, reproduction and biological condition of the European flat

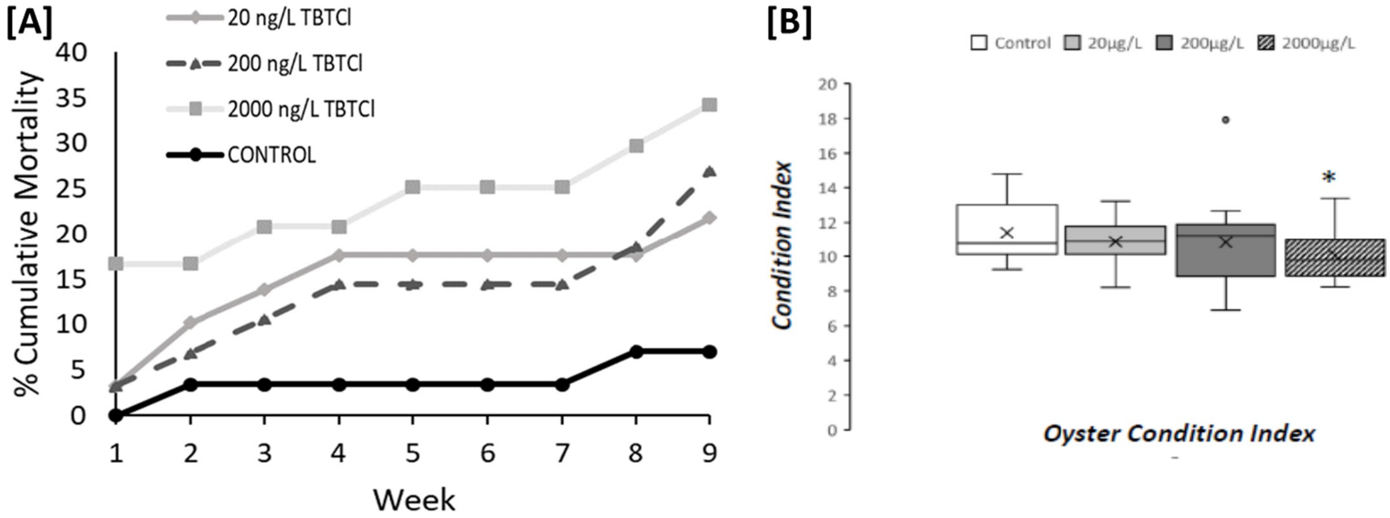

Fig 8. Cumulative mortality (A) and Body Condition index (B) of *Ostrea edulis* treated with 20 ng/L (n = 30), 200 ng/L (n = 30), 2000 ng/L of TBTCl (n = 30), and a negative control (n = 30) during 9 weeks.

https://doi.org/10.1371/journal.pone.0280777.g008

oyster *Ostrea edulis*, both as a model species and as a particular species of focus for habitat restoration internationally. The digestive gland has been proposed as a reliable target-tissue for exploring the effects of pollutants at cellular, biochemical, and molecular levels due to its capacity to reflect metabolism and biotransformation of xenobiotics [55].

Through targeted metabolomics we recorded a decrease in tissues ATP concentrations associated with an increase of ADP concentration, identifying this as an indicator of metabolic stress [56]. It has previously been reported that the mitochondrial F_0F_1 complex is a target of TBTCl toxicity in mussel digestive gland mitochondria, preventing ATP formation by impeding the proton flux that drives ATP synthesis from ADP and Pi [25, 57]. Given the direct inhibitory effect of TBT noted for Mg and Ca-ATPase and associated with osmoregulation [58–60]. TBT may directly affect solute transport through inhibition of ATPase activity and decreasing tissue substrate concentrations, principally ATP.

The TCA cycle appears to be one important mechanism affected after chronic exposure to TBTCl (Fig 3). This disruption occurred at multiple points in TCA cycle when oysters were exposed at the highest TBT concentration. All TBTCl doses significantly decreased tissue fumarate levels ($p<0.001$ for all exposures; Fig 3E) indicating impairment of the succinate dehydrogenase step of TCA cycle. Moreover, the decrease in fumarate levels indicates a break in the TCA cycle represented as a decrease in the contribution of the succinate dehydrogenase step to facilitate active proton shuttling against the concentration gradient. TBTCl exposure was also associated with a depletion of oxaloacetate ($p<0.001$) (Fig 3G) following exposure to the highest dose of TBTCl; this was unexpected given the preservation of malate levels in oyster tissue. One possible explanation may be the diversion of oxaloacetate towards pyruvate metabolism through pyruvate carboxylase. However, given that metabolomic analysis measures only 'steady-state' metabolite levels and not rates of substrate flux, we are unable to test these hypotheses. In addition, the reduction in fumarate levels and oxaloacetate [61] supports the idea that disruption of the TCA cycle may have contributed to the decline in ATP levels following exposure to TBT; however, we cannot exclude the possibility that TBT can act as an uncoupling agent to dissipate the mitochondrial proton gradient at the F_0F_1 complex [62, 63]. This may be partially supported by the dose-dependent nature of the decrease in ATP levels.

Free fatty acids represent a highly-efficient source of metabolic energy, in terms of ATP produced per molecule of substrate [64]. The decline in tissue levels of palmitate, oleate, stearate and linoleate may further reflect the recruitment of substrate to support the synthesis of ATP. Together these observations imply that oysters maintain food intake in the presence of TBT, but that this was insufficient to sustain the oxidative metabolism to support the production of ATP.

It has been reported that organotins can trigger apoptosis via the mitochondrial pathway by blocking mitochondrial ATP synthesis, loss of mitochondrial membrane integrity and increasing the oxidative stress by promoting ROS production [65]. Oxidative stress occurs when there is an imbalance between the production of free radicals and the cells ability to efficiently remove them [66–68]. Reduced glutathione (GSH) plays a key role in the detoxification of a large number of xenobiotics, and it has been reported alongside other antioxidant enzymes in marine invertebrates as part of the antioxidant defences [69–71]. TBT has shown an oxidative stress effect caused by inhibition of this enzyme in rat cell cultures [72], fish [73, 74] and oysters (Fig 5, this paper, see also [75]). In this last instance it was suggested that lower levels of GSH in TBT-exposed oysters can be explained by the conjugation of GSH with the antifouling agent [75]. This could imply that after a prolonged environmental exposure to TBT, *O. edulis* could face a GSH depletion and a subsequent reduction on the detoxification capacity which can make these individuals more vulnerable to the oxidative stress. A mismatch between the

cellular ATP demand and mitochondrial ATP generation, an increase in ROS production and the lack of ability to efficiently remove them can result in energy deficiency [56].

The release of important second messengers and stress signals such as ROS and Ca^{2+} as a result of mitochondrial stress are involved in the regulation of the cellular signalling cascades including AMP kinase, and other stress-responsive kinases that ultimately lead to upregulation of the antioxidant defense [56]. However, recently a potential role for AMP-activated protein kinase in gametogenesis with sex-specific regulation has been described for the hermaphrodite Pacific oyster (*Magallana gigas*) [76]. The authors reported *AMPKα* mRNA and protein levels were significantly higher in male oysters compared with female at the same stage of gonad development [76]. Moreover, whilst *AMPKα* expression in female oysters was absent late in gametogenesis, for male oysters the expression on AMPKα protein levels increased throughout gamete development [76]. Given that AMPKα controls post-translational modification of a range of processes within the oyster [77], can be activated in response to environmental stress by pollutants [78], and may play a role in gonad development [76], our data suggest that the declining ATP/AMP ratio may further contribute to the changes observed for gonads and male:female ratio of native oysters exposed to TBTCl (Fig 7).

It is clear that high concentrations of TBTCl are associated with an arrest in gonadal maturation and an increase in mortality in this species, but the environmentally relevant concentrations used in this study (20 ng/L and 200 ng/L of TBTCl) showed an effect in sex determination as well. A female-skewed trend was observed in the control which can be explained by the possibility of investing in the production of female gametes, that can be more energetically costly than the production of male gametes, when oysters have been fed with an algal diet rich in carbohydrates [79]. In contrast, a higher proportion of males found at these concentrations of TBTCl (Fig 7) could indicate that organotin compounds have a masculinizing effect in *O. edulis*. These findings might, in part, explain the bias towards males reported in some natural populations of *O. edulis* [40]. The Solent (United Kingdom) fishery has reported several collapses in *O. edulis* populations during the last century [30–35], and significant reductions in the number of brooding female-phase oysters and a biased sex ratio towards male-phase oyster [40].

Previous studies suggested a correlation of observed effect in molluscs and retinoid X receptor (RXR) interaction with TBT [80, 81]. Furthermore, the RXR/PPAR heterodimer has been suggested as the specific target for TBT [81]. We identify perturbations in the 9/13-cis-retinoic acid metabolites in oysters exposed to increasing concentrations of TBTCl, in particular a dose-dependent increase in 9/13-cis-retinoic acid concentration in digestive gland tissues associated with exposure to increasing TBTCl. Binding studies with the rockshell, *Tritonia clavigera*, showed that TBT binds to the RXR with high affinity [80] and it has further been shown that 9-cis retinoic acid induces imposex in females of *Nucella lapillus* to the same degree as tributyltin when administered at similar concentrations (1 μg/g body weight) [82]. Collectively, our observations combined with earlier studies suggest an alternative option to TBT-related effects and a novel mechanism for organotin-induced toxic and reproductive effects in invertebrates that require further exploration.

Digestive tissue lipid and protein concentrations increased in oysters exposed to TBTCl (Fig 6). Tributyltin has been shown to be a potent inducer of adipogenesis in vertebrates [83] and can also act as a potent inducer of lipid and fatty acid accumulation in gastropod snails [84]. It has been reported that TBT exposure can influence the reproductive functions of fish through a lipotoxic mechanism [85]. These authors showed that exposure of rockfish (*Sebastiscus marmoratus*) to TBT for 48 d induces a lipid accumulation response in the ovaries of this species, showing an increase of interstitial ectopic lipid accumulation and total lipids. An increase in lipid accumulation and altered fatty acid homeostasis were also observed in the digestive gland/

gonad complex in the ramshorn snail (*Marisa cornuarietis*) exposed to 125 and 500 ng/L TBT [84]. The accumulation of excess lipids in nonadipose tissues leading to metabolic disorders has been reported [86]. There is no evidence of an adverse effect of lipids accumulation in gonadal tissues of oysters but it is a key issue that requires further investigation. In addition, the excessive accumulation of lipids in nonadipose tissues can exceed the cell's capacity to store or use them generating a lipotoxicity response, which is characterized by destruction of organelle membranes and the activation of stress pathways that can lead to apoptosis [87].

We conclude that although gonad tissue developed in the visceral mass of all oysters, there was limited evidence for maturation of sexually mature organs, evidenced by increased proportion of stages G0 and G1 in most oysters exposed to TBTCl, especially at 2000 ng/l (Fig 7). The absence or limited differentiation of lipid-rich gonadal tissue in *O. edulis* exposed to high concentrations (2.6 μg/L) of TBT was also observed by Thain [24] after 75 days of exposure confirming that elevated levels of this compound cause an apparent failure in gonad differentiation in this species. The increase in stages G0 and G1 was also observed for oysters treated with 20 and 200 ng/L of TBTCL in this study, although the proportion was lower. The gonadal tissue is the main organ that varies throughout the reproductive cycle in bivalves [88], so it could be expected that in those oysters in which the gonad failed to develop (stages G0 and G1) there was an overall impact to body condition (Fig 8B). Gonadal development is an important parameter that provides a useful indication about the reproductive status and activity at individual and population levels [89, 90] but is an energy-consuming process [79]. Energy reserves and lipid synthesis are processes required for gonadal maturation so it can be expected that animals would use these reserves to complete gametogenesis [38, 79, 94]. During gametogenesis, glycogen stored in the gonad is broken down into glucose to produce ATP, NADPH, and NADH which are necessary for the synthesis of other organic compounds including fatty acids and nucleic acids [91]. With the reduction of ATP molecules, a lack of energy available to undergo gametogenesis successfully could be expected and so hence the decrease in reaching maturity. It has been suggested that reproductive processes may be compromised under stressful conditions in an attempt to devote more energy toward survival [92]. In the same manner, in mussels reproduction can be compromised to conserve energy that increases changes of survival under stressful conditions and adverse environments [93]. It could be expected that under stress and a reduction of ATP molecules available *O. edulis* may allocate energy from reproduction towards survival as observed at the highest concentration of TBT used in this study.

Sperm cells require less energy than oocytes to complete maturation, so it could be expected that mature females allocate more energy per unit organ to mantle-gonads than mature males showing differences in energy allocation between sexes during gonadal maturation [79]. This has also been observed in *Aulacomya atra* and *Scrobicularia plana* males and females showing that even when they can reach a similar energy content of the mantle-gonad, they use this energy in a different way: males have gonads of larger size but with lower energy per unit of mass than females [79, 94]. Environmental pollutants could create an additional stress that, in addition to the reduction of ATP molecules and enough energy to initiate gametogenesis, could stimulate the production of male gametes in a hermaphroditic species such as *O. edulis*.

We demonstrate that TBT exposure at environmentally relevant concentrations has the potential to be toxic and have effects on different process related to the production of energy, metabolism, affecting both reproduction and ultimately the survival of *Ostrea edulis* exposed to this compound. Numerous restoration efforts for *O. edulis* and its habitat are in progress in UK and Europe and restoration of other ostreid and crassostreid species is being pursued globally. However, our data suggest that it is necessary to consider pollution by organotin

compounds in water and sediments in areas close to ports and areas influenced by shipping or other industrial activities.

Conclusion

TBTCl has been widely recognized as an endocrine disruptor in gastropods. In this study, 20ng/l and 200ng/l TBTCl caused a masculinization effect evidenced by the increase of male oysters under these treatments at the end of the trial. At the highest concentration, an increase in oysters classified as inactive and no males were found. These alterations in gonad maturation were associated with other metabolic impacts to the exposed oysters, including a decrease tissue ATP concentration through combination of the disruption of the TCA cycle and fatty acid metabolite concentrations, the down-regulation of molecules involved in the TCA cycle, an increase in tissue lipid and protein content. In addition, a reduction in the antioxidant response supports the idea of toxicity through the imposition of oxidative damage, triggered by exposure to TBT toxicity in this species. Ultimately, these processes were associated with disruptions to the normal process of gonad maturation, a reduction in body condition, and an increase in mortality over the duration of the experiment. The persistence of environmental pollutants, such as TBTCl, could cause an additional threat to the declining *Ostrea edulis* populations and related taxa around the world by increasing mortality, changing metabolic responses, reproductive maturation and skewing sex-ratios in natural populations, thereby threatening current efforts to restore these species in coastal waters.

Materials & methods

Oysters

Ostrea edulis (5–7 cm at their maximum diameter) were obtained from Galway Bay in Ireland in January 2018 and were transferred to the National Oceanography Centre Southampton (NOCS) where they were acclimated for four weeks. During the acclimation period, they were placed in seawater tanks (about 1L/oyster) with continuous aeration at the same temperature (8˚C) as at the grow-out site (https://www.seatemperature.org/europe/ireland/gaillimh.htm). Oysters were fed *ad libitum* daily with 40000 cells/ml of a live mixed algae diet (40% *Tetraselmis suecica*, 40% *Pavlova lutheri* and 20% *Phaedactylum tricornutum*). The research was approved by written consent of the University of Southampton Faculty of Environmental and Life Science Ethics Committee, under the University's Ethics and Research Governance Policy.

TBT treatments

After the acclimation process and before starting the exposure experiments, 6 oysters were taken as a control and these data are referred to as initial time (t0). One-hundred and twenty oysters were divided randomly among four treatment tanks (n = 30 per treatement): 20 ng/L Tributyiltin chloride (TBTCl), 200 ng/L TBTCl 2000 ng/L TBTCl and a negative control. TBTCl (Sigma–Aldrich; Stenheim, Germany) dissolved in 100% ethanol, which was then dried under a nitrogen stream and diluted 1:100 with 1-μm filtered sterilized seawater to give a 0.5mg/mL stock solution. Processes such as adsorption of TBT into the wall of the glass containers and/or microbial degradation can occur, so the water was renewed and spiked with TBTCl every 48h (renewal volume 80% of total aquarium water). The 20 and 200 ng/l treatments were designed to mimic environmentally relevant values and have been reported in seawater samples taken from the Solent [11]. The highest concentration tested in this study exceeded environmental levels but it has been shown that TBT can be bioaccumulated in

bivalves causing tissue concentrations much higher than in environment [95–97]. By 2009, mean TBT concentrations in seawater (4.5–10 ng/L as Sn), sediment (0.02 μg/g as Sn) and biota were reported [11]. TBT is strongly adsorbed to organic matter and sediments retain concentrations of TBT for long periods and it can be released to the water column when sediment is disturbed, for example by dredging which happens regularly on a very large scale in the Solent [1, 2, 5]. Remaining TBT in Southampton water could also reflect some residual TBT on small boats and continuing influence of large vessels in Southampton Water.

All the treatments were kept at 10˚C; previous experiments have shown that two months of exposure at that temperature resulted in a slower but competent gonadal maturation [98]. Water temperatures were controlled throughout the experiments using a free-standing chiller unit (TECO, model TR60). The salinity, pH, temperature, conductivity and dissolved oxygen were measured in every aquarium at least twice per week.

At the beginning of the experiment 6 individuals were selected to determine sex and gametogenic stage. After 9 weeks of exposure, individuals (n = 13 per treatment) were randomly selected and sacrificed. Then gonadal tissues were fixed in Bouin's solution for histological analysis or kept at -20˚C for biochemical analysis.

The temperature of the exposure experiment was maintained at 10˚C because previous experiments have shown that two months of exposure at that temperature resulted in a slower but competent gonadal maturation [98].

Biological indices

Measurements of height (H), length (L), width (Wi; all to 0.01mm. Shell cavity volume (SVol), Fresh tissue weight (FW) and total weight (W, to the nearest 0.1g) were measured. After removal of the shells, the Condition Index (CI) was calculated for each bivalve: ([total fresh tissue weight/total weight] x 100) [99, 100].

Histological analysis

Transverse tissue sections 5 mm thick parallel to the anterior–posterior axis were sampled from each oyster for histological examination following a standard protocol [89, 90]. The samples were dehydrated through an ethanol series (70%, 80%, 90% and dehydrated ethanol) overnight for each concentration. The samples were embedded in paraffin, and the wax blocks were sectioned at 6-μm using a rotary microtome (*Leitz Wetzler*, *model 1212*), and stained with hematoxlyn/eosin (Cellpath Ltd) [89, 90]. Because maturation is not a homogenous process and female and male gametes can be present in different follicles at different maturation stages at the same time [101–104], three slides per animal were prepared from three different sections separated by 500 μm to determine sex and developmental stage of the gonad. Sex was recorded as indeterminate (I), female solely (F), male solely (M), hermaphrodite with both sexes equally represented (HBS), hermaphrodite predominantly male (HPM) and hermaphrodite predominantly male (HPF) according to da Silva et al. [39]). The gametogenic stage of the gonad was identified as inactive (G0), early gametogenesis (G1), advanced gametogenesis (G2), ripe gonad (G3), partially spawned gonad (G4) and reabsorbing gonad (G5) adopted by da Silva et al. [39].

Energy reserves

Energy reserves (lipids, carbohydrates and proteins) were quantified in the gonad of each animal. After homogenization by hand in liquid nitrogen with a porcelain mortar and pestle, 1mg of the fine powder obtained was then homogenized with a motorised grinder with 1000 uL of distilled deionized water. The samples were analysed in duplicate. From this initial

homogenate 300 μL were mixed well with 100 μL of distilled water and a mixture of methanol/chloroform (2:1, v/v) [105] following a gravimetric method suggested by Mann and Gallager [106]. Cholesterol (95%, ACROS Organics™) was used for method calibration. Recoveries were reported at 90% for cholesterol every time the method was carried out. Carbohydrate and protein assay began with extraction of the initial water homogenate (500 μL) with trichloracetic acid to give a final concentration of 5% w/v after mixing [106]. The carbohydrate content of the supernatant was assayed by the phenol-sulphuric acid method of Raymont et al. [107] using glucose (D-glucose anhydrous, analytical grade, Fisher Scientific) as a standard. The total protein content in the precipitate was measured using a Bicinchoninic Acid Protein Assay Kit (BCA). The calculation of total protein, lipid, and carbohydrate was based on the dry tissue weight (DW) of each individual and determined as a percentage (%).

Metabolomic profile of TBT-exposed *Ostrea edulis*

The visceral mass tissue section (0.1 g) was obtained such that the dorsal-ventral aspect passes through the digestive gland and gill tissue just posterior to the palps. Samples were immediately placed into liquid nitrogen and stored at -80˚C until further analysis. To characterize the metabolic changes that occurred in *O. edulis* exposed to different steroid types and concentrations, the metabolomic profiles of all the animals in each treatment was carried out by the McCullagh Metabolomics Laboratory for untargeted metabolomics, Department of Chemistry at the University of Oxford [54]. In brief, each sample was analysed using up to three separate liquid chromatography with tandem mass spectrometry (LC-MS/MS) methods using two different LC systems (Thermo Scientific ICS-5000+ ion chromatography system and a Thermo Ultimate 3000). Each was coupled directly to a Q-Exactive HF Hybrid Quadrupole-Orbitrap mass spectrometer with a HESI II electrospray ionisation source (Thermo Scientific, San Jose, CA). The IC-MS/MS was performed using a ICS-5000+ HPLC system incorporating an electrolytic anion generator (KOH) which was programmed to produce a OH–gradient prior to MS analysis (Thermo Scientific Dionex AERS 500). The C18 reversed-phase analysis of underivatised samples was performed using a Thermo Utimate 3000 UHPLC system with a gradient elution program coupled directly to a Q-Exactive HF Hybrid Quadrupole-Orbitrap mass spectrometer. And the third LC-MS method used a sample derivatisation protocol followed by analysis based on a modified version of the Waters AccQ-Tag method [108]. C18 reversed-phase analysis of derivatised samples was also performed using the Thermo Ultimate 3000 UHPLC system coupled directly to a Q-Exactive HF Hybrid Quadrupole-Orbitrap mass spectrometer. For data processing, ion species were identified with reference to an 'in-house' database created from authenticated standards. Briefly, pure compounds were purchased from chemical suppliers (e.g. Sigma-Aldrich, UK; Tocris UK; Tokyo Chemicals Industry, UK). These standards were then diluted in appropriate solvent (80% methanol) and separated chromatographically by different methods. Each compound was then examined using QExactive Mass Spectrometer (Thermo, UK). Each authenticated standard was identified by collection of discrete data: this included chromatographic retention time; accurate mass (5 decimal places), compound fragmentation hence allowing the identification if different structural isomers with reference to differing fragmentation and retention characteristics. Raw data files were processed using ProgenesisQI (Waters, Elstree, UK). This process included alignment of retention times, peak picking by identification of the presence of natural abundance isotope peaks, characterising multiple adducts forms and identification of metabolites using our in-house database. Retention times, accurate mass values, relative isotope abundances and fragmentation patterns were compared between authentic standards and the samples measured. Identifications were accepted only when the following criteria were met: $<$5ppm differences between

measured and theoretical mass (based on chemical formula), <30 seconds differences between authenticated standard and analyte retention times, isotope peak abundance measurements for analytes were >90% matched to the theoretical value generated from the chemical formula. Where measured, fragmentation patterns were matched to at least the base peak and two additional peak matches in the MS/MS spectrum to within 12ppm.

Statistical analysis

The normality of data and homogeneity of variances were evaluated using the Shapiro Wilk and the Levene's tests, respectively. The assumptions of parametric tests were not met, so non-parametric tests were applied. The Kruskal-Wallis H-test was used to determine differences in mortality, biometric parameters (W, H, Wi, SVol, FW, CI), biochemical variables (lipids, carbohydrates, proteins) and gonadal development. When non-parametric Kruskal and Wallis test was significant, differences were then evaluated using a non-parametric the Mann and Whitney test. Spearman's correlation was used to test the relationship between biochemical variables (lipids, carbohydrates, proteins). Chi-square statistics were used to test sex ratios against a 1:1 ratio. Fisher's exact test was used to compare mortality between treatments. Metabolomics results between the treatments and the control were analysed using univariate statistical analysis determining fold-change and t-tests between experimental groups for compound features and combined in volcano plots (FDR-adjusted p-values should be reported). PCA and PLS-DA were also used to analyse the patterns in metabolomic profiles among treatments. Data is presented as MEAN ±SEM. Statistical significance was assigned at $p \leq 0.05$.

Supporting information

S1 Fig. Principal component analysis and partial least square discriminant analysis model comparing *Ostrea edulis* metabolomic profiles among TBTCl treatments: 20 ng/L (n = 13), 200 ng/L (n = 13) and 2000 ng/L (n = 13), and a negative control (n = 13) after 9 weeks of exposure. IC-MS/MS was performed using a ICS-5000+ HPLC system, C18 reversed-phase analysis of underivatised samples was performed using a Thermo Utimate 3000 UHPLC system, and C18 reversed-phase analysis of derivatised samples was also performed using the Thermo Utimate 3000 UHPLC system coupled directly to a Q-Exactive HF Hybrid Quadrupole-Orbitrap mass spectrometer. Models are assessed by cross validation (using R2, Q2 and Accuracy; R2 should be higher than Q2 which should be >0.4 and within 0.3 if the R2 values, the closer to 1 the R2 and Q2 the strong the model). Partial Least Squares-Discrimination Analysis (PLSDA) indicated a high degree of correlation between the modelled data and the predicted analysis for TBTCl exposure. Furthermore, permutation test analysis indicated that the results were significantly different ($p<0.01$ for all) implying the data were not overfitted.
(DOCX)

S1 Table. *Ostrea edulis* biometric parameters (mean±SD) for total Weight (W), Height (H), length (L), width (Wi), Shell volume (SVol), flesh weight (FW) and Condition Index (CI) at the beginning of the experiment (t0), under different treatments (20 ng/L, 200 ng/L and 2000 ng/L of TBTCl), and a negative control after 9 weeks.
(DOCX)

S2 Table. Gonad development stages classified as inactive (G0), early gametogenesis (G1), advanced gametogenesis (G2), ripe gonad (G3), partially spawned gonad (G4) and reabsorbing gonad (G5) according to the sex categories (females and males) identified at the end of the exposure to TBTCl.
(DOCX)

S1 File.
(ZIP)

Acknowledgments

We gratefully acknowledge help from the aquarium technician Robbie Robinson for his technical assistance for the maintenance of oysters in the National Oceanography Centre Southampton.

Author Contributions

Conceptualization: Lina M. Zapata-Restrepio.

Formal analysis: Lina M. Zapata-Restrepio, David Hauton.

Funding acquisition: Malcolm D. Hudson, Ian D. Williams.

Investigation: Lina M. Zapata-Restrepio.

Methodology: Lina M. Zapata-Restrepio, David Hauton.

Project administration: Malcolm D. Hudson.

Resources: Chris Hauton, Malcolm D. Hudson, Ian D. Williams, David Hauton.

Supervision: Chris Hauton, Malcolm D. Hudson, Ian D. Williams.

Validation: Lina M. Zapata-Restrepio, David Hauton.

Visualization: Lina M. Zapata-Restrepio, David Hauton.

Writing – original draft: Lina M. Zapata-Restrepio.

Writing – review & editing: Lina M. Zapata-Restrepio, Chris Hauton, Malcolm D. Hudson, Ian D. Williams, David Hauton.

References

1. Gadd GM. Microbial interactions with tributyltin compounds: detoxification, accumulation, and environmental fate. Sci Total Environ. 2000 Aug 21; 258(1–2):119–27. https://doi.org/10.1016/s0048-9697(00)00512-x PMID: 11007284
2. Pynaert K, Speleers L. Development of an integrated approach for the removal of tributyltin (TBT) from waterways and harbors: Prevention, treatment and reuse of TBT contaminated sediments. Belgium. 2000. Available at: http://www.vliz.be/imisdocs/publications/78032.pdf (Last accessed 30 June 2022).
3. Matthiessen P. Detection, monitoring, and control of tributyltin-an almost complete success story. Environ Toxicol Chem. 2013 Mar 1; 32(3):487–9. https://doi.org/10.1002/etc.2108 PMID: 23418041
4. Clark EA, Sterritt RM, Lester JN. The fate of tributyltin in the aquatic environment. Environ Sci Technol. 1988 Jun; 22(6):600–4.
5. Dowson PH, Bubb JM, Lester JN. Persistence and Degradation Pathways of Tributyltin in Freshwater and Estuarine Sediments. Estuar Coast Shelf Sci. 1996 May 1; 42(5):551–62.
6. Ayanda OS, Fatoki OS, Adekola FA, Ximba BJ. Fate and Remediation of Organotin Compounds in Seawaters and Soils. Chem Sci Trans. 2012 Aug 25; 1(3):470–81.
7. Seligman PF, Valkirs AO, Lee RF. Degradation of tributyltin in San Diego Bay, California, waters. Environ Sci Technol. 1986 Dec; 20(12):1229–35.
8. Alzieu C. Environmental impact of TBT: The French experience. Sci Total Environ. 2000; 258(1–2):99–102. https://doi.org/10.1016/s0048-9697(00)00510-6 PMID: 11007281
9. Cleary JJ. Organotin in the marine surface microlayer and subsurface waters of south-west England: Relation to toxicity thresholds and the UK environmental quality standard. Mar Environ Res. 1991 Jan 1; 32(1–4):213–22.

10. Waite ME, Waldock MJ, Thain JE, Smith DJ, Milton SM. Reductions in TBT concentrations in UK estuaries following legislation in 1986 and 1987. Mar Environ Res. 1991 Jan 1; 32(1–4):89–111.
11. Langston WJ, Pope ND, Davey M, Langston KM, O' Hara SCM, Gibbs PE, et al. Recovery from TBT pollution in English Channel environments: A problem solved? Mar Pollut Bull. 2015; 95(2):551–64. https://doi.org/10.1016/j.marpolbul.2014.12.011 PMID: 25733081
12. Oliveira DD de, Rojas EG, Fernandez MA dos S. Should TBT continue to be considered an issue in dredging port areas? A brief review of the global evidence. Ocean Coast Manag. 2020; 197:105303.
13. Cassi R, Tolosa I, Mora S de. A survey of antifoulants in sediments from Ports and Marinas along the French Mediterranean coast. Mar Pollut Bull. 2008; 56(11):1943–8. https://doi.org/10.1016/j.marpolbul.2008.07.011 PMID: 18793783
14. Carić H, Klobučar G, Štambuk A. Ecotoxicological risk assessment of antifouling emissions in a cruise ship port. J Clean Prod. 2016; 121(2016):159–68.
15. Solent European Marine Sites (SEMS). Annual Management Report. 2019. Available at: http://www.solentems.org.uk/sems/AMR/SEMS_2019%20SSG_Comments.pdf (Last accessed 30 June 2022).
16. Hsia MP, Liu SM. Accumulation of organotin compounds in Pacific oysters, *Crassostrea gigas*, collected from aquaculture sites in Taiwan. Sci Total Environ. 2003; 313(1–3):41–8.
17. Meng PJ, Wang JT, Liu LL, Chen MH, Hung TC. Toxicity and bioaccumulation of tributyltin and triphenyltin on oysters and rock shells collected from Taiwan maricuture area. Sci Total Environ. 2005; 349 (1–3):140–9. https://doi.org/10.1016/j.scitotenv.2005.01.016 PMID: 16198676
18. Yang R, Zhou Q, Jiang G. Butyltin accumulation in the marine clam Mya arenaria: An evaluation of its suitability for monitoring butyltin pollution. Chemosphere. 2006 Mar 1; 63(1):1–8. https://doi.org/10.1016/j.chemosphere.2005.07.074 PMID: 16188291
19. Chen C, Chen L, Huang Q, Chen Z, Zhang W. Organotin contamination in commercial and wild oysters from China: Increasing occurrence of triphenyltin. Sci Total Environ. 2019; 650:2527–34. https://doi.org/10.1016/j.scitotenv.2018.09.310 PMID: 30293005
20. Higuera-Ruiz R, Elorza J. Shell thickening and chambering in the oyster *Crassostrea gigas*: Natural and anthropogenic influence of tributyltin contamination. Environ Technol. 2011; 32(6):583–91.
21. Ruiz JM, Bryan GW, Gibbs PE. Chronic toxicity of water tributyltin (TBT) and copper to spat of the bivalve *Scrobicularia plana*: Ecological implications. Mar Ecol Prog Ser. 1994; 113(1–2):105–18.
22. Axiak V, Sammut M, Chircop P, Vella A, Mintoff B. Laboratory and field investigations on the effects of organotin (tributyltin) on the oyster, *Ostrea edulis*. Sci Total Environ. 1995; 171(1–3):117–20.
23. Héral M, Alzieu C, Deslous-Paoli J. Effect of organotin compounds (TBT) used in antifouling paints on cultured marine molluscs—a literature study. Aquac Biotechnol Prog. 1989;1081–9.
24. Thain JE. Toxicity of TBT To Bivalves: Effects on Reproduction, Growth and Survival. Ocean Conf Rec. 1986;1306–13.
25. Nesci S, Ventrella V, Trombetti F, Pirini M, Pagliarani A. The mitochondrial F1FO-ATPase desensitization to oligomycin by tributyltin is due to thiol oxidation. Biochimie. 2014; 97(1):128–37.
26. Khondee P, Srisomsap C, Chokchaichamnankit D, Svasti J, Simpson RJ, Kingtong S. Histopathological effect and stress response of mantle proteome following TBT exposure in the Hooded oyster *Saccostrea cucullata*. Environ Pollut. 2016; 218:855–62.
27. Perry F, Jackson A. Native oyster (*Ostrea edulis*). In Tyler-Walters H. and Hiscock K. (eds). MarLIN—Mar Life Inf Netw Biol Sensit Key Inf Rev [on-line]. 2017.
28. Laing I, Walker P, Areal F. A Feasibility Study of Native Oyster (Ostrea Edulis) Stock Regeneration in the United Kingdom. CARD Project FC1016 Oyster Stock Regeneration -A Review of Biological, Technical and Economic Feasibility. 2005. Available at: https://www.seafish.org/document/?id=2976D1CB-0216-444E-A719-3F7EF69F51FB (Last accessed 30 June 2022).
29. Harding S, Nelson L, Glover T. Solent Oyster Restoration Project. 2016. Available at: https://www.bluemarinefoundation.com/wp-content/uploads/2015/11/20160525_Solent-Oyster-Restoration-Project_Management-Plan_Final-version.pdf (Last accessed 30 June 2022).
30. Smith IP, Low PJ, Moore PG. Legal aspects of conserving native oysters in Scotland. Mar Pollut Bull. 2006; 52(5):479–83. https://doi.org/10.1016/j.marpolbul.2006.03.005 PMID: 16631812
31. FAO. Global aquaculture production: Fisheries and Aquaculture Departament. 2019. Available at: http://www.fao.org/fishery/statistics/global-aquaculture-production/query/es (Last accessed 30 June 2022).
32. Tubbs CR. The ecology, conservation and history of the Solent. Packard; 1999. 184 p.
33. Key D, Davidson PE. A review of the development of the Solent oyster fishery 1972–80. Technical report. 1981. Available at: https://www.cefas.co.uk/publications/lableaflets/lableaflet52.pdf (Last accessed 30 June 2022).

34. Gravestock V, James F, Goulden M. Solent Native Oyster (*Ostrea edulis*) Restoration: Literature Review & Feasibility Study. 2014.

35. Southern Inshore Fisheries and Conservation Authority (IFCA). Solent Oyster Fishery 2018 Stock Survey Report. 2018. Available at: https://www.southern-ifca.gov.uk/solent-dredge-permit (Last accessed 30 June 2022).

36. Eagling LE. Reproductive success of the re-laid native oyster Ostrea edulis in Chichester harbour. MSci Dissertation, University of Southampton, England. 2012.

37. Acarli S, Lök A, Kirtik A, Acarli D, Serdar S, Kucukdermenci A, et al. Seasonal variation in reproductive activity and biochemical composition of flat oyster (*Ostrea edulis*) in the Homa Lagoon, Izmir Bay, Turkey. Sci Mar. 2015 Dec 30; 79(4):487–95.

38. Hassan M, Qin JG, Li X. Gametogenesis, sex ratio and energy metabolism in *Ostrea angasi*: implications for the reproductive strategy of spermcasting marine bivalves. Malacol Soc London J Molluscan Stud J Molluscan Stud. 2018; 84:38–45.

39. da Silva PM, Fuentes J, Villalba A. Differences in gametogenic cycle among strains of the European flat oyster *Ostrea edulis* and relationship between gametogenesis and bonamiosis. Aquaculture. 2009; 287(3–4):253–65.

40. Kamphausen LM, Jensen AC, Hawkins L. Unusually High Proportion of Males in a Collapsing Population of Commercially Fished Oysters (*Ostrea edulis*) in the Solent, United Kingdom. J Shellfish Res. 2011 Aug 30; 30(2):217–22.

41. Baeza JA, Braga AA, López-Greco LS, Perez E, Negreiros-Fransozo ML, Fransozo A. Population dynamics, sex ratio and size at sex change in a protandric simultaneous hermaphrodite, the spiny shrimp *Exhippolysmata oplophoroides*. Mar Biol. 2010; 157(12):2643–53.

42. Lewis C, Santos EM. Physiological impacts of chemcial pollutants in marine animals. In: Solan M, Whiteley NM, editors. Stressors in the marine environment: physiological and ecological responses; societal implications. Oxford, GB.: Oxford University Press,; 2016. p. 384.

43. Blaber SJM. The occurrence of a penis-like outgrowth behind the right tentacle in spent females of *Nucella lapillus* (L.). J Molluscan Stud. 1970 Dec 1; 39(2–3):231–3.

44. Gibbs PE. A Male genital defect in the dog-whelk, *Nucella lapillus* (Neogastropoda), favouring survival in a TBT-polluted area. J Mar Biol Assoc United Kingdom. 1993 Aug 11; 73(3):667–78.

45. Ketata I, Denier X, Hamza-Chaffai A, Minier C. Endocrine-related reproductive effects in molluscs. Comp. Biochem. Physiol. C Toxicol. Pharmacol. 2008; 147(3):261–270. https://doi.org/10.1016/j.cbpc.2007.11.007 PMID: 18282745

46. Matthiessen P, Gibbs PE. Critical appraisal of the evidence for tributyltin-mediated endocrine disruption in mollusks. Environ Toxicol Chem. 1998 Jan 1; 17(1):37–43.

47. Horiguchi T, Kojima M, Kaya M, Matsuo T, Shiraishi H, Morita M, et al. Tributyltin and triphenyltin induce spermatogenesis in ovary of female abalone, *Haliotis gigantea*. Mar Environ Res. 2002; 54(3–5):679–84.

48. Thain JE, Waldock MJ. The Impact of Tributyl Tin (TBT) Antifouling Paints on Molluscan Fisheries. Water Sci Technol. 1986 Apr 1; 18(4–5):193–202.

49. Roberts M, Bender M, De Lisle P, Sutton H, Williams R. Sex Ratio and Gamete Production in American Oysters Exposed to Tributyltin in the Laboratory. In: OCEANS '87. IEEE; 1987. p. 1471–6.

50. Bennema FP, Engelhard GH, Lindeboom H. *Ostrea edulis* beds in the central North Sea: delineation, ecology, and restoration. ICES J Mar Sci. 2020; 77(7–8):2694–705.

51. Choi JY, Hong GH, Ra K, Kim KT, Kim K. Magnetic characteristics of sediment grains concurrently contaminated with TBT and metals near a shipyard in Busan, Korea. Mar Pollut Bull. 2014; 85(2):679–85. https://doi.org/10.1016/j.marpolbul.2014.03.029 PMID: 24703766

52. Norén A, Karlfeldt Fedje K, Strömvall AM, Rauch S, Andersson-Sköld Y. Integrated assessment of management strategies for metal-contaminated dredged sediments–What are the best approaches for ports, marinas and waterways? Sci Total Environ. 2020; 716:135510. https://doi.org/10.1016/j.scitotenv.2019.135510 PMID: 31837871

53. Fathollahzadeh H, Kaczala F, Bhatnagar A, Hogland W. Significance of environmental dredging on metal mobility from contaminated sediments in the Oskarshamn Harbor, Sweden. Chemosphere. 2015; 119:445–51. https://doi.org/10.1016/j.chemosphere.2014.07.008 PMID: 25084063

54. Walsby-Tickle J, Gannon J, Hvinden I, Bardella C, Abboud MI, Nazeer A, et al. Anion-exchange chromatography mass spectrometry provides extensive coverage of primary metabolic pathways revealing altered metabolism in IDH1 mutant cells. Commun Biol. 2020; 3(1):1–12.

55. Faggio C, Tsarpali V, Dailianis S. Mussel digestive gland as a model tissue for assessing xenobiotics: An overview. Sci Total Environ. 2018; 636:220–9. https://doi.org/10.1016/j.scitotenv.2018.04.264 PMID: 29704717

56. Sokolova I. Mitochondrial adaptations to variable environments and their role in animals' stress tolerance. Integr Comp Biol. 2018; 58(3):519–31. https://doi.org/10.1093/icb/icy017 PMID: 29701785

57. Nesci S, Ventrella V, Trombetti F, Pirini M, Borgatti AR, Pagliarani A. Tributyltin (TBT) and dibutyltin (DBT) differently inhibit the mitochondrial Mg-ATPase activity in mussel digestive gland. Toxicol Vitr. 2011; 25(1):117–24. https://doi.org/10.1016/j.tiv.2010.10.001 PMID: 20950683

58. Pagliarani A, Bandiera P, Ventrella V, Trombetti F, Pirini M, Nesci S, et al. Tributyltin (TBT) inhibition of oligomycin-sensitive Mg-ATPase activity in mussel mitochondria. Toxicol Vitr. 2008; 22(4):827–36. https://doi.org/10.1016/j.tiv.2007.12.015 PMID: 18261881

59. Ventrella V, Nesci S, Trombetti F, Bandiera P, Pirini M, Borgatti AR, et al. Tributyltin inhibits the oligomycin-sensitive Mg-ATPase activity in *Mytilus galloprovincialis* digestive gland mitochondria. Comp Biochem Physiol—C Toxicol Pharmacol. 2011; 153(1):75–81.

60. Nesci S, Ventrella V, Trombetti F, Pirini M, Pagliarani A. Tributyltin-driven enhancement of the DCCD insensitive Mg-ATPase activity in mussel digestive gland mitochondria. Biochimie. 2012; 94(3):727–33. https://doi.org/10.1016/j.biochi.2011.11.002 PMID: 22108212

61. Scrutton MC. Fine control of the conversion of pyruvate (phosphoenolpyruvate) to oxaloacetate in various species. FEBS Lett. 1978; 89(1):1–9.

62. Nesci S, Ventrella V, Trombetti F, Pirini M, Pagliarani A. Tributyltin (TBT) and mitochondrial respiration in mussel digestive gland. Toxicol Vitr. 2011; 25(4):951–9. https://doi.org/10.1016/j.tiv.2011.03.004 PMID: 21396439

63. Bragadin M, Marton D, Manente S, Scutari G, Toninello A. Tributyltin and mitochondria: New evidence in support of an uncoupling mechanism and a further characterisation of the transport mechanism. Inorganica Chim Acta. 2003; 348:225–8.

64. Livingstone DR. Invertebrate and vertebrate pathways of anaerobic metabolism: evolutionary considerations. J Geol Soc London. 1983; 140(1):27–37.

65. Jaksic Z. Mechanisms of Organotin-Induced Apoptosis. In: Biochemical and Biological Effects of Organotins. Bentham Science Publishers; 2012. p. 149–63.

66. Simon H-U, Haj-Yehia A, Levi-Schaffer F. Role of reactive oxygen species (ROS) in apoptosis induction. Apoptosis. 2000; 5(5):415–8. https://doi.org/10.1023/a:1009616228304 PMID: 11256882

67. Elmore S. Apoptosis: a review of programmed cell death. Toxicol Pathol. 2007 Jun; 35(4):495–516. https://doi.org/10.1080/01926230701320337 PMID: 17562483

68. Hongmei Z. Extrinsic and Intrinsic Apoptosis Signal Pathway Review. In: Apoptosis and Medicine. InTech; 2012. Available at: https://www.intechopen.com/chapters/38236 (Last accessed 30 June 2022).

69. Gamble SC, Goldfarb PS, Porte C, Livingstone DR. Glutathione peroxidase and other antioxidant enzyme function in marine invertebrates (*Mytilus edulis*, *Pecten maximus*, *Carcinus maenas* and *Asterias rubens*). Mar Environ Res. 1995 Jan 1; 39(1–4):191–5.

70. Lee RF. Glutathione S-transferase in marine invertebrates from Langesundfjord. Mar Ecol—Prog Ser. 1988; 46:33–6.

71. Belcheva NN, Chelomin VP. Glutathione S-transferase activity in marine invertebrates from Peter the Great Bay in the Sea of Japan. Russ J Mar Biol. 2011 Jan 5; 37(1):62–8.

72. Ishihara Y, Kawami T, Ishida A, Yamazaki T. Tributyltin induces oxidative stress and neuronal injury by inhibiting glutathione S-transferase in rat organotypic hippocampal slice cultures. Neurochem Int. 2012 Jun; 60(8):782–90. https://doi.org/10.1016/j.neuint.2012.03.004 PMID: 22449404

73. Li Z-H, Li P, Shi Z-C. Responses of the hepatic glutathione antioxidant defense system and related gene expression in juvenile common carp after chronic treatment with tributyltin. Ecotoxicology. 2015 Apr 13; 24(3):700–5. https://doi.org/10.1007/s10646-014-1416-2 PMID: 25582114

74. Wang C, Zhao Y, Zheng R, Ding X, Wei W, Zuo Z, et al. Effects of tributyltin, benzo[a]pyrene, and their mixture on antioxidant defense systems in Sebastiscus marmoratus. Ecotoxicol Environ Saf. 2006 Nov; 65(3):381–7. https://doi.org/10.1016/j.ecoenv.2005.08.003 PMID: 16197995

75. Park MS, Kim YD, Kim BM, Kim YJ, Kim JK, Rhee JS. Effects of antifouling biocides on molecular and biochemical defense system in the gill of the pacific oyster *Crassostrea gigas*. PLoS One. 2016; 11(12):1–20.

76. Guévélou E, Huvet A, Galindo-Sánchez CE, Milan M, Quillien V, Daniel JY, et al. Sex-specific regulation of AMP-activated protein kinase (AMPK) in the pacific oyster *Crassostrea gigas*. Biol Reprod. 2013; 89(4):1–15.

77. Epelboin Y, Quintric L, Guévélou E, Boudry P, Pichereau V, Corporeau C. The kinome of Pacific oyster *Crassostrea gigas*, its expression during development and in response to environmental factors. PLoS One. 2016; 11(5):1–24.

78. Goodchild CG, Frederich M, Zeeman SI. AMP-activated protein kinase is a biomarker of energetic status in freshwater mussels exposed to municipal effluents. Sci Total Environ. 2015; 512–513:201–9. https://doi.org/10.1016/j.scitotenv.2015.01.065 PMID: 25622267

79. Pérez AF, Boy CC, Curelovich J, Pérez-Barros P, Calcagno JA. Relationship between energy allocation and gametogenesis in *Aulacomya atra* (Bivalvia: Mytilidae) in a sub-Antarctic environment. Rev Biol Mar Oceanogr. 2013 Dec; 48(3):459–469.

80. Nishikawa JI, Mamiya S, Kanayama T, Nishikawa T, Shiraishi F, Horiguchi T. Involvement of the Retinoid X Receptor in the Development of Imposex Caused by Organotins in Gastropods. Environ Sci Tchnol. 2004; 38:6271–6. https://doi.org/10.1021/es049593u PMID: 15597881

81. Vogeler S, Galloway TS, Isupov M, & Bean TP. Cloning retinoid and peroxisome proliferator-activated nuclear receptors of the Pacific oyster and in silico binding to environmental chemicals. PloS one. 2017; 12(4),e0176024. https://doi.org/10.1371/journal.pone.0176024 PMID: 28426724

82. Castro LFC, Lima D, Machado A, Melo C, Hiromori Y, Nishikawa J, et al. Imposex induction is mediated through the Retinoid X Receptor signalling pathway in the neogastropod *Nucella lapillus*. Aquat Toxicol. 2007 Nov 15; 85(1):57–66.

83. Grün F, Watanabe H, Zamanian Z, Maeda L, Arima K, Cubacha R, et al. Endocrine-disrupting organotin compounds are potent inducers of adipogenesis in vertebrates. Mol Endocrinol. 2006; 20(9):2141–55. https://doi.org/10.1210/me.2005-0367 PMID: 16613991

84. Janer G, Navarro JC, Porte C. Exposure to TBT increases accumulation of lipids and alters fatty acid homeostasis in the ramshorn snail *Marisa cornuarietis*. Comp Biochem Physiol C Toxicol Pharmacol. 2007 Sep; 146(3):368–74.

85. Zhang J, Zuo Z, Xiong J, Sun P, Chen Y, Wang C. Tributyltin exposure causes lipotoxicity responses in the ovaries of rockfish, *Sebastiscus marmoratus*. Chemosphere. 2013 Jan 1; 90(3):1294–9.

86. Weinberg JM. Lipotoxicity. Kidney Int. 2006 Nov; 70(9):1560–6. https://doi.org/10.1038/sj.ki.5001834 PMID: 16955100

87. Brookheart RT, Michel CI, Schaffer JE. As a Matter of Fat. Cell Metab. 2009 Jul; 10(1):9–12. https://doi.org/10.1016/j.cmet.2009.03.011 PMID: 19583949

88. Filgueira R, Comeau LA, Landry T, Grant J, Guyondet T, Mallet A. Bivalve condition index as an indicator of aquaculture intensity: A meta-analysis. Ecol Indic. 2013 Feb 1; 25:215–29.

89. Kim Y, Ashton-Alcox KA, Powell EN. Histological Techniques for Marine Bivalve Molluscs: Update. Silver Spring, MD. NOAA Technical Memorandum NOS NCCOS 27. Vol. 76. 2006.

90. Howard DW, Lewis EJ, Keller BJ, Smith CS. Histological techniques for marine bivalve mollusks and crustaceans, 2nd edition. 2004;218.

91. Wang C. Roles of Sex Steroids in reproduction of the Sea Scallop. Dalhousie University, Halifax, Nova Scotia; 2000. Available at: http://citeseerx.ist.psu.edu/viewdoc/download?doi=10.1.1.427.3208&rep=rep1&type=pdf (Last accessed 30 June 2022).

92. Wingfield JC, Sapolsky RM. Reproduction and resistance to stress: When and how. J Neuroendocrinol. 2003; 15(8):711–24. https://doi.org/10.1046/j.1365-2826.2003.01033.x PMID: 12834431

93. Petes LE, Menge BA, Harris AL. Intertidal Mussels Exhibit Energetic Trade-Offs between Reproduction and Stress Resistance. Ecol Monogr. 2008; 78(3):387–402.

94. Mouneyrac C, Linot S, Amiard JC, Amiard-Triquet C, Métais I, Durou C, et al. Biological indices, energy reserves, steroid hormones and sexual maturity in the infaunal bivalve *Scrobicularia plana* from three sites differing by their level of contamination. Gen Comp Endocrinol. 2008; 157(2):133–41.

95. Langston W., Burt G., Mingjiang Z. Tin and organotin in water, sediments, and benthic organisms of Poole Harbour. Mar Pollut Bull. 1987 Dec 1; 18(12):634–9.

96. Anderson RS., Unger MA, Burreson EM. Enhancement of Perkinsus marinus disease progression in TBT-exposed oysters (*Crassostrea virginica*). Mar. Environ. Res. 1996; 42(1–4):177–180.

97. Bryan GW, Gibbs PE, Burt GR, Hummerstone LG. The effects of tributyltin (TBT) accumulation on adult dog-whelks, *Nucella lapillus*: long-term field and laboratory experiments. J mar biol Ass UK. 1987; 67:525–44.

98. Zapata-Restrepo LM, Hauton C, Williams ID, Jensen AC, Hudson MD. Effects of the interaction between temperature and steroid hormones on gametogenesis and sex ratio in the European flat oyster (*Ostrea edulis*). Comp Biochem Physiol -Part A Mol Integr Physiol. 2019; 236(March):110523.

99. Walne PR. Experiments on the culture in the sea of the butterfish *Venerupis decussata* L. Aquaculture. 1976 Aug 1; 8(4):371–81.

100. Lawrence DR, Scott GI. The Determination and Use of Condition Index of Oysters. Estuaries. 1982 Mar; 5(1):23.

101. Korringa P. Recent Advances in Oyster Biology. Q Rev Biol. 1952 Sep; 27(3):266–308. https://doi.org/10.1086/399023 PMID: 13004275

102. Sparck R. Studies of the biology of the oyster (*Ostrea edulis*) in Limfjord, with special reference to the influence of temperature and the sex-change. Rep Dan Biol Sta. 1925; 30:1–84.

103. Coe WR. Histological basis of sex changes in the american oyster (*Ostrea virginica*). Science (80-). 1932 Aug 5; 76(1962):125–7.

104. Loosanoff VL. Gametogenesis and spawning of the European oyster, *O. edulis*, in waters of Maine. Biol Bull. 1962 Feb; 122(1):86–94.

105. Bligh EG, Dyer WJ. A rapid method of total lipid extraction and purification. Can J Biochem Physiol. 1959 Aug; 37(8):911–7. https://doi.org/10.1139/o59-099 PMID: 13671378

106. Mann R, Gallager SM. Physiological and biochemical energetics of larvae of *Teredo navalis* L. and *Bankia gouldi* (Bartsch) (Bivalvia: Teredinidae). J Exp Mar Bio Ecol. 1985 Feb; 85(3):211–28.

107. Raymont JEG, Austin J, Linford E. Biochemical Studies on Marine Zooplankton: I. The Biochemical Composition of Neomysis integer. ICES J Mar Sci. 1964 Mar 1; 28(3):354–63.

108. Salazar C, Armenta JM, Cortés DF, Shulaev V. Combination of an AccQ·Tag-Ultra Performance Liquid Chromatographic Method with Tandem Mass Spectrometry for the Analysis of Amino Acids. In: Alterman M, Hunziker P (eds) Amino Acid Analysis Methods in Molecular Biology (Methods and Protocols), vol 828. Humana Press, Totowa, NJ; 2012.
